# Asthma Prevalence Among Adults in Qassim Region, Saudi Arabia

**DOI:** 10.7759/cureus.53229

**Published:** 2024-01-30

**Authors:** Faisal Alamer, Ahmed S Almuzaini, Sami Alharbi, Marya Algoblan, Faisal Alayed, Rayan A Alsaqri, Yazeed S Alsweed

**Affiliations:** 1 College of Medicine, Qassim University, Buraydah, SAU; 2 Internal Medicine and Pulmonology, King Fahad Specialist Hospital, Buraydah, SAU

**Keywords:** general internal medicine, prevalence, pulmonary disease, saudi arabia, qassim region, asthma

## Abstract

Background: Asthma is a major non-communicable disease affecting both children and adults and is the most common chronic disease among children. It has a significant effect on patient's daily lives, as well as a big economic impact on society, as it affects 262 million people of the population globally. According to the previous research conducted in the Aseer region in southwestern Saudi Arabia, the prevalence rate of bronchial asthma was found to be 19.2%. Also, a number of studies revealed a significant prevalence of asthma in Saudi Arabia. Therefore, it is simple and effective to measure specific asthma symptoms among the adult population by utilizing the European Community Respiratory Health Survey (ECRHS) criteria.

Objectives: To investigate asthma prevalence and measure asthma symptoms among Saudi adults in Qassim, Saudi Arabia using the ECRHS.

Methods: This cross-sectional study targets the males and females living in the Qassim region of Saudi Arabia. The study was conducted by handing out a validated, self-administered questionnaire among adult male and female patients in the Qassim region of Saudi Arabia. Both descriptive and inferential statistics analyses were conducted. To test the association, both chi-square and Fisher’s exact tests were carried out. For the test, a p-value less than 0.05 was considered statistically significant.

Results: The study involved 461 participants who met the inclusion criteria. The study found that 137 (29.9%) participants reported having tightness in their chests when they woke up. Among the respondents who reported these symptoms were 83 (60.1%) female and 55 (39.9%) male respondents. This shows a statistically significant difference between the gender and severity of chest tightness upon waking up (p = 0.008) with more females experiencing it than the male gender. More so, there was a statistically significant difference between the gender and severity of shortness of breath (p = 0.008), with more females (81, 60.4%) having the symptoms than the male gender (53, 39.6%). In addition, the study results reveal statistically significant gender differences among the patients who were diagnosed with asthma by the physician (p = 0.003), with more males (51, 65.4%) having been diagnosed than the female gender (27, 34.6%). Asthma attacks in the 12 months (p = 0.047) and the use of tobacco products (p = 0.001) were also found to have a statistically significant difference across the genders. This was shown by most males (26, 65.0%) who had asthma attacks in the 12 months and 70 (98.6%) who smoked any tobacco products than the few females (14, 35.0%) who reported to have had asthma attacks in the 12 months and one (1.4%) who reported smoking any tobacco products.

Conclusion: This study noted that the prevalence of asthma symptoms varied based on the gender of the participants. Therefore, the study suggests that gender should be taken as an important factor while evaluating the severity and how asthma-related symptoms manifest.

## Introduction

Asthma is a common condition due to chronic inflammation of the lower respiratory tract characterized by symptoms of shortness of breath, cough, chest tightness, and/or wheezing [1,2]. Its pathogenesis is complicated by the involvement of numerous cell types and inflammatory mediators. Th2 lymphocytes are often the mediating factor in airway inflammation; their cytokine secretion causes mast cell activation, eosinophilia, leukocytosis, and increased synthesis of immunoglobulin E (IgE) by B cells [2]. There are two types of asthma: intermittent and persistent. Persistent asthma can be mild, moderate, or severe. Some patients progress from having intermittent asthma to having persistent asthma. Additionally, patients with asthma can be categorized as having cough variant asthma, aspirin-exacerbated respiratory disease, allergic (mediated by IgE), nonallergic (often triggered by viral upper respiratory tract infections or no apparent cause), occupational, exercise-induced, and potentially fatal [3]. The primary objective of therapy for asthma is to achieve minimum or no symptoms, normal sleep and activity patterns, and optimal lung function as a result of the disease. With patient education, avoiding environmental triggers, customized medication, and routine follow-up, this kind of control can be achieved [4]. Asthma is frequently accompanied by a variety of comorbidities. Rhinitis, sinusitis, gastric reflux disease, obstructive sleep apnea, hormone disorders, and psychopathologies are the most commonly reported asthma concomitant problems [4].

Several studies have assessed the prevalence of asthma in Saudi adults, including a 2016 cross-sectional survey in Riyadh with 2,405 participants using the European Community Respiratory Health Survey (ECRHS) questionnaire, reporting an 18.2% prevalence of wheezing without a cold in the past 12 months [5]. Another study conducted in Riyadh secondary schools found lifetime wheeze, wheeze in the past 12 months, and physician-diagnosed asthma rates of 25.3%, 18.5%, and 19.6%, respectively, among 3,073 students [6]. In the Aseer region, a study on 960 adults using the International Study of Asthma and Allergies in Childhood (ISAAC) questionnaire reported a 19.2% prevalence rate of bronchial asthma [7]. In Rabigh City, a survey using the ISAAC questionnaire found prevalence rates of 31.5% for physician-diagnosed asthma, 23.5% for any wheezing, and 14.9% for wheezing in the past 12 months among children/adolescents [8]. Additionally, a 2022 study on 7,955 adults from 20 regions in Saudi Arabia reported an overall asthma symptom prevalence of 14.2% [9]. Different methodologies were used in the study, with the Saudi Health Interview Survey (SHIS, 2013) reporting a lower national prevalence rate of 4.1% based on doctor-diagnosed asthma [10]. According to the findings of a comprehensive analysis of 31 studies conducted in different areas of Saudi Arabia, the pooled weighted prevalence of asthma was determined to be 14.3%. The regional variance for asthma ranged from 3.1% in Qassim to 33.7% in Al-Hofuf [11]. Another systematic review of 12 studies on pediatric asthma in different Saudi Arabian cities and regions revealed varying prevalence rates, with the highest in Al-Hofuf (33.7%) and the lowest in Abha (9%) [12]. The objective of the present study is to assess the prevalence of asthma and specific asthma symptoms among the adult Saudi population residing in the Qassim region of Saudi Arabia, utilizing the ECRHS questionnaire.

## Materials and methods

Study design, duration, and setting

This cross-sectional, questionnaire-based study was conducted among males and females aged between 22 and 44 years living in the Qassim region of Saudi Arabia from November 2023 to January 2024.

Sample size and sampling technique

The Ministry of Interior reports that the estimated population of the Qassim region is 1,016,765. By employing the Raosoft sample size calculator (Raosoft Inc., Seattle, WA), it was determined that 385 participants were required for this investigation to achieve a 95% confidence interval, a 5% margin of error, and a predetermined prevalence of 50%. The participants were enlisted utilizing a convenient sampling method, and their informed consent was obtained. The participants were duly apprised of the research objectives prior to obtaining their informed consent.

Inclusion criteria

Inclusion criteria included individuals living in the Qassim region, 20-44 years old, literate in Arabic or English, and willing to participate.

Data collection tool

Following a comprehensive explanation of the research objectives, the participants were administered an online self-administered questionnaire that had been validated and translated into Arabic by a previous study to collect the data [5]. The questionnaire consists of three sections. The first section explains the research objectives and the consent for participation, the second section is for collecting the sociodemographic data (like living in the Qassim region or not, gender, and age), and the third section includes the ECRHS questionnaire.

Data analysis

Data were collected and entered in Excel spreadsheets (Microsoft Corporation, Redmond, WA) for data cleaning. After data cleaning, data were coded for statistical analysis. SPSS version 26 (IBM Corp., Armonk, NY) was used for all data analyses. Categorical variables were presented using counts and percentages. The chi-squared test was used to determine the association between categorical variables. Statistical significance was defined as a p-value of 0.05 or less.

Ethical considerations

The research was conducted after obtaining the required approvals from the Al-Qassim Research Ethics Committee (QERC), Qassim province (H-04-Q-001).

## Results

Table 1 shows the general characteristics of the study participants. The study's inclusion criteria were met by 461 participants. There were 233 female (50.5%) and 228 male (49.5%) participants. The participants' ages ranged from 20 to 40 years. In addition, 424 (92.0%) of the participants had completed tertiary education, while 37 (8.0%) had only completed secondary or lower education. In the previous 12 months, 92 (20.0%) of the participants reported wheezing or whistling in the absence of a cold, 137 (29.7%) reported wheezing or whistling within the last 12 months, 138 (29.9%) reported waking up with their chest constricted, and 134 (29.1%) reported difficulty breathing. Furthermore, 175 individuals (38.0%) stated that they were awakened by a coughing fit. Regarding medical history, 78 participants (16.9%) reported having a medical professional diagnose them with asthma, 40 (8.7%) reported having an asthma attack within the previous year, and 29 (6.3%) were currently on medication for their condition. Among the participants, 192 (41.6%) reported having allergies in their noses. Finally, 71 individuals (15.4%) disclosed using tobacco products.

**Table 1:** General characteristics of the participants (N = 461). Data have been presented as n (%).

Variable	Characteristic	N (%)
Gender	Male	228 (49.5%)
	Female	233 (50.5%)
Age	20-44 years	461 (100%)
Education level	Secondary and lower	37 (8.0%)
	Tertiary	424 (92.0%)
Wheezing or whistling within the last 12 months	Yes	137 (29.7%)
	No	324 (70.3%)
Wheezing or whistling without cold	Yes	92 (20.0%)
	No	369 (80.0%)
Woken up with chest tightness	Yes	138 (29.9%)
	No	323 (70.1%)
Woken up by shortness of breath	Yes	134 (29.1%)
	No	327 (70.9%)
Woken up by an attack of coughing	Yes	175 (38.0%)
	No	286 (62.0%)
Physician-diagnosed asthma	Yes	78 (16.9%)
	No	383 (83.1%)
Asthma attack in the last 12 months	Yes	40 (8.7%)
	No	421 (91.3%)
Currently taking any medicine for asthma	Yes	29 (6.3%)
	No	432 (93.7%)
Nasal allergies	Yes	192 (41.6%)
	No	269 (58.4%)
Smoke any tobacco products	Yes	71 (15.4%)
	No	390 (84.6%)

Table 2 shows that 138 (29.9%) individuals said they woke up with tightness in their chest, with 83 (60.1%) females and 55 (39.9%) males, respectively, reporting having this symptom. The association was statistically significant at p = 0.008. Similarly, 134 participants (29.1%) reported that they had experienced dyspnea upon waking up; this symptom was reported by more females (81, 60.4%) than males (53, 39.6%), and a p-value of 0.008 was obtained, indicating a statistically significant association. Moreover, 78 (16.9%) participants reported physician-diagnosed asthma, and there was a significant difference between the genders, in particular, more men (51, 65.4%) than women (27, 34.6%) reported having an asthma diagnosis from a doctor (p = 0.003). In addition, 40 (8.7%) participants said they had experienced an asthma attack within the previous 12 months; males experienced an attack at a higher rate than females, with 26 (65.0%) males reporting an attack versus 14 (35.0%) females (p = 0.047). Nevertheless, 71 (15.4%) individuals disclosed using tobacco products, showing a noteworthy distinction between males (70, 98.6%) and females (1, 1.4%), with a p-value of 0.001.

**Table 2:** Prevalence and severity of asthma symptoms among the participants by gender. Data have been presented as n (%). * P-value < 0.05 was considered statistically significant.

Symptoms	Total (n = 461) (%)	Male (n = 228) (%)	Female (n = 233) (%)	P-value*
Wheezing or whistling within the last 12 months				0.919
Yes	137 (29.7%)	67 (51.1%)	70 (48.9%)	
No	324 (70.3%)	161 (49.7%)	161 (50.3%)	
Wheezing or whistling without cold				0.418
Yes	92 (20.0%)	49 (45.5%)	43 (46.5%)	
No	369 (80.0%)	179 (48.5%)	190 (51.5%)	
Woken up with chest tightness				0.008
Yes	138 (29.9%)	55 (39.9%)	83 (60.1%)	
No	323 (70.1%)	173 (53.6%)	150 (46.4%)	
Woken up by shortness of breath				0.008
Yes	134 (29.1%)	53 (39.6%)	81 (60.4%)	
No	327 (70.9%)	175 (53.5%)	152 (46.5%)	
Woken up by an attack of coughing				0.633
Yes	175 (38.0%)	84 (48.0%)	91 (52.0%)	
No	286 (62.0%)	144 (49.7%)	142 (50.3%)	
Physician-diagnosed asthma				0.003
Yes	78 (16.9%)	51 (65.4%)	27 (34.6%)	
No	383 (83.1%)	177 (46.2%)	206 (53.8%)	
Asthma attack in the last 12 months				0.047
Yes	40 (8.7%)	26 (65.0%)	14 (35.0%)	
No	421 (91.3%)	202 (48.0%)	219 (52.0%)	
Currently taking any medicine for asthma				0.342
Yes	29 (6.3%)	17 (58.6%)	12 (41.4%)	
No	432 (93.7%)	211 (48.8%)	221 (51.2%)	
Nasal allergies				0.108
Yes	192 (41.6%)	86 (44.8%)	106 (55.2%)	
No	269 (58.4%)	142 (52.8%)	127 (47.2%)	
Smoke any tobacco products				0.001
Yes	71 (15.4%)	70 (98.6%)	1 (1.4%)	
No	390 (84.6%)	158 (40.5%)	232 (59.5%)	

Table 3 shows that there was no statistical significance between the gender of the participants and the wheezing or whistling within the last 12 months. In addition, there was no statistical significance in the education level of the participants and wheezing or whistling within the last 12 months. All p-values were greater than 0.05.

**Table 3:** Association between sociodemographic characteristics and wheezing within the last 12 months Data have been presented as n (%). * P-value < 0.05 was considered statistically significant.

Characteristics	N (%)	P-values*
Gender		
Male	228 (49.5%)	0.919
Female	233 (50.5%)	
Education		0.457
Secondary and lower	37 (8.0%)	
Tertiary	424 (92.0%)	

## Discussion

This study focused on investigating the prevalence of asthma and measuring symptoms of asthma among Saudi adults in Qassim, Saudi Arabia. Based on the findings of the study, 92 (20.0%) of the participants reported wheezing or whistling in the absence of a cold, and 137 (29.7%) reported wheezing or whistling within the last 12 months. On the other hand, 138 (29.9%) of the participants reported waking up with their chest constricted, and 134 (29.1%) reported difficulty breathing. Moreover, 175 (38.0%) respondents indicated that they were awakened by a coughing fit. On matters concerning the medical history of the participants, 78 (16.9%) of the respondents reported having a medical professional diagnose them with asthma, 40 (8.7%) reported having an asthma attack within the previous year, and 29 (6.3%) were currently on medication for their condition. Additionally, 192 (41.6%) participants reported having allergies in their noses. A total of 71 individuals (15.4%) indicated that they use tobacco products. In consistence with 6.3% of the participants revealed in this study undergoing asthmatic condition medication, a study by Dodge et al. noted that the percentage of patients with asthma was 6.6%, with the highest rates found in pediatric patients. Furthermore, among older subjects, rates were relatively high, with the majority having concurrent diagnoses of "chronic bronchitis and/or emphysema." Other types of wheezing were very common in this population sample; point prevalence rates of wheezing exceeded 30% in the majority of age groups [13]. Additionally, in a study by Croisant, it was revealed that among the participants evaluated from around 70 countries, the prevalence rate of asthma was found to be 4%, which is close to 6.3% obtained in this study [14]. Another study conducted in Australia revealed that among the participants who were evaluated, 27.4% of them had difficulties in breathing when they woke up, which is closely similar (29.1%) to this study [15].

Further, the study revealed that 138 (29.9%) individuals said they woke up with tightness in their chest, with 83 (60.1%) females and 55 (39.9%) men, respectively, who reported having this symptom. The difference across the genders was statistically significant at p = 0.008. The current prevalence of asthma attacks was 11.2% in the previous year; overall, the prevalence of asthma was 15.1%. The study found that smoking currently presents a significant risk of developing asthma. As noted in a study by Rönnebjerg et al., there was a statistically significant difference in the prevalence of asthma among the participants surveyed across the gender (p < 0.000). The study also reported variation in the asthmatic symptoms reported based on the gender of the participants with some symptoms revealing more in female gender than male and vice versa [16].

Similarly, 134 participants (29.1%) reported that they had experienced dyspnea upon waking up; this symptom was reported by more females (81, 60.4%) than males (53, 39.6%), respectively, with a p-value of 0.008 indicating a statistically significant association. Wong et al. noted in their study that about 49% of students had a persistent cough, and 33.5% said they occasionally woke up in the middle of the night due to wheezing. However, the difference in the symptoms reported was statistically significant across the genders of the participants [17]. A study by Saglan et al. indicated that higher rates of asthma symptoms such as shortness of breath were reported by more female students than male students who participated in the study; hence, the findings complement the findings obtained in this study [18].

Moreover, the study revealed that 78 (16.9%) participants reported physician-diagnosed asthma, and there was a significant difference between the genders, in particular, more men (51, 65.4%) than women (27, 34.6%) reported having asthma diagnosis from a doctor (p = 0.003). In addition, 40 (8.7%) participants indicated that they had experienced an asthma attack within the previous 12 months; males experienced an attack at a higher rate than females, with 26 (65.0%) males reporting an attack versus 14 (35.0%) females, with a p-value of 0.047. Nevertheless, 71 individuals (15.4%) disclosed using tobacco products, showing a noteworthy distinction between males (70, 98.6%) and females (1, 1.4%), with a p-value of 0.001. As noted in other studies, without accounting for comorbidities, the prevalence of asthma among the participants varied, with more females being affected by the symptoms than males. However, among the smokers, males were found to be more affected by asthma symptoms than the female gender [19,20].

Some of our study's shortcomings may be attributed to its design. This study, like all others with a cross-sectional design, cannot deduce causality. Another limitation of this study is that it relied on self-reports of asthma diagnosed by a physician to determine the presence of asthma. However, as previously stated, in epidemiological studies, self-reported asthma that has been medically diagnosed is typically used. Despite these limitations, the study's methodology and the use of random selection give the results more weight.

## Conclusions

The study concludes that there is a statistically significant difference between the genders in regard to waking up with symptoms like tightness in the chest and shortness of breath. Compared to male participants, female participants were more likely to have these symptoms. The study also noted asthma diagnoses varied based on gender, with a higher diagnosis rate being found in men than in women. In addition, there were variations in the prevalence of tobacco use and asthma attacks, with more men than women reporting these conditions in both cases, respectively. Therefore, this study suggests that gender should be taken as an important factor while evaluating the severity and how asthma-related symptoms manifest.
